# Is Evaluation of Hepatitis A Immunity Required or Not?

**DOI:** 10.5812/kowsar.1735143X.764

**Published:** 2011-12-20

**Authors:** Gholam Ali Ghorbani

**Affiliations:** 1 Department of Infectious Diseases, Baqiyatallah Research Center for Gastroenterology and Liver Diseases, Tehran, IR Iran; 2 Health Research Center, Baqiyatallah University of Medical Sciences, Tehran, IR Iran

**Keywords:** Hepatitis A Virus, Antibiotic Prophylaxis, Epidemiology

## Abstract

Hepatitis A virus (HAV) infection is one of the most common causes of acute hepatitis and it is a serious health problem worldwide. HAV infection is a vaccine preventable disease that can produce the lifelong immunity seen in many developed countries with the vaccination schedule administered to children; however this vaccine is not used in developing countries at the present time. Improvements in food and water hygiene have caused a displacement of hepatitis A infection from children to adults which has increased mortality rates. Therefore evaluation of HAV immunity levels can help health authorities develop polices for prophylaxis especially in developing countries.

The hepatitis A virusis a small non-enveloped RNA virus of the Picornaviridae family that causes more than 1.5 million cases of hepatitis throughout the world, and it is one of the most common causes of acute hepatitis as well as being an important worldwide health problem according to Vilibic-Cavlek et al. [1]. Although Croatia appears to be a low prevalence HAV infection region, it is similar to other developed countries so the risk of a HAV outbreak is high and prophylaxis should be considered because community immunity is correspondingly low and prone to outbreaks [1]. Although evaluation of immunity is important in developing countries it seems that a study of HAV immunity in Croatia is not warranted and universal vaccination of children against HAV infection is more cost effective than an evaluation of immunity, the opposite was found in a study of HAV in Iran [2]. On the other hand, nearly all of the older age groups in developing countries are immune to HAV and therefore are not prone to HAV infections, this relates to a study by Tanjana et al. By contrast in Iran the immunity of children to HAV is higher than in Croatia and universal vaccination is not as cost effective as an evaluation of people's immunity especially those in close contact with the disease, then prophylaxis especially with immune globulin is indicated [1][2][3].

Hepatitis A virus is transmitted through fecal material with the most common route being orally and outbreaks can occur in closed populations which have crowded living conditions [4]. In developing countries improvements in both hygiene and the sanitation of food and water have caused a decrease in the prevalence of HAV infections similar to that of developed countries producing low levels of HAV immunity in their children. A study by Tanjana et al. showed that immunity levels in children under 15 years of age have reduced from 18.7% to 5.6% so in the near future all children in the world will progress to non-immune levels of HAV, therefore public health managers should keep vaccination of children in mind [1][5][6]. In most geographical regions men are at higher risk than women of contracting HAV infections because they have higher occupational risks including unpleasant work involving contaminated sewage and more men work outside of the home and the food and water that they eat may be unhealthy don't resemble to study of tanjana, so the use of prophylaxis for HAV infections is more important for men [7][8].

The highest number of asymptomatic HAV infection outbreaks occurred in developing countries while symptomatic sporadic infections were seen in developed countries [9][10]. HAV infections are often acute and relief from symptoms occurs in a short time but sometimes prolonged or relapsing hepatitis is induced. The clinical spectrum of HAV infection ranges from asymptomatic to fulminant hepatitis with complications and atypical manifestations that may be life threatening in adulthood and the elderly [9][11]. In a study by Vilibic-Cavlek et al. who also showed this subject and therefore paradoxically symptomatic hepatitis A infections were not seen in developing countries because more infections occur in children and vice versa symptomatic HAV infections are seen more often in developed countries due to the increased numbers of infections occurring in adults [12]. This study agreed with Vilibic-Cavlek et al. that evaluating immunity in children could determine the best time for vaccination. Due to improved living conditions in recent years immunity against HAV is diminishing so both developed and developing countries are at high risk for this infection, like Croatia, particularly in children [6][13].

Hepatitis A infection is a vaccine preventable disease, this vaccine can induce lifelong immunity and is used in many developed countries, but this vaccine is not yet readily available in some developing countries [7][8][13]. The vaccine has a high level of immunogenicity that in some cases reaches 100% sero-protection levels and it can control outbreaks in overcrowded, susceptible populations [13]. The two main ways that HAV can be prevented is by passive immunoprophylaxis and active immunoprophylaxis through vaccination [3]. Passive prophylaxis can be done with immune globulin for a short time pre- and post-exposure to the hepatitis A virus and it can be used any time that a vaccine is not available. Centers for Disease Control and Prevention (CDC) also currently advocates that vaccine or immune globulin can be used as a post-exposure prophylaxis [13][14]. With exposure of a person to the virus whether they are suspected to be immune or not, a prophylaxis can be given to provide immunity to the HAV [2][8]. Moreover, immunoglobulin is effective as a vaccine but it is not approved for general prevention purposes, therefore for mass prophylaxis vaccination is advocated [13]. In developing countries such as Iran following improvements in food and water hygiene, children's immunity has reduced, therefore hepatitis A vaccination should be considered and an immune survey should be done every few years to help health managers decide when to provide universal vaccination against the HAV [14][15]. Epidemiology measures that produce information about HAV infection levels in different parts of the world is important for health authorities in order to provide prevention policies of HAV infection rates especially in developing countries [16].

## Conclusion

Ultimately improvements in the hygiene of food and water have caused the displacement of HAV infections from children to adult populations which has increased the mortality rate. Therefore in conformity with the advice from Vilibic-Cavlek et al. children in developed countries warrant evaluation of their immunity levels as well as those in developing countries until health authorities can suggest a time for prophylaxis. However in developed countries because the seroprevalence of HAV infections is much lower than in children of developing countries, it seems that vaccination against the HAV should be considered for all children.
